# Bone Shortening After Amputation: A Report of Two Cases

**DOI:** 10.7759/cureus.41024

**Published:** 2023-06-27

**Authors:** Yohei Tanaka, Takaaki Ueno, Toshiki Miura

**Affiliations:** 1 Rehabilitation Medicine, JR Tokyo General Hospital, Tokyo, JPN; 2 Orthopedic Surgery, JR Tokyo General Hospital, Tokyo, JPN

**Keywords:** bone length of stump, bone shortening, stump length, stump, amputation

## Abstract

We report two cases of shortening of the bone at the stump after amputation. Case 1 was a 57-year-old male with a traumatic transhumeral amputation. The remaining humerus had shortened by 3.5 cm in eight months. Case 2 was a 27-year-old male with a traumatic transtibial amputation. The remaining tibia had shortened by 1.4 cm in 72 months. These two cases had the same cause of amputation, but the amputation site, age, and time course differed. Few studies have examined the bone length of stumps after amputation. The bone length of stumps is generally assumed to not change after amputation. However, the residual bone at the stump can shorten after amputation.

## Introduction

The stump is swollen and has a large volume in the early postoperative period. Subsequently, the stump volume gradually decreased over time. This phenomenon is called the maturation of the stump [1-3]. Stump maturation occurs owing to edema reduction and muscle atrophy in the stump [1]. However, no previous reports support the relationship between stump maturation and shortening of the residual bone at the stump.

As the bone of the stump shortens, the stump becomes even smaller compared with changes in the soft tissue alone. The shorter the stump, the more difficult for the prosthetist to fit a prosthetic socket into the patient's stump. The fit of the prosthetic socket directly affects the ease of prosthesis use. In other words, shorter stumps are more likely to lead to worse prosthetic socket fit and subsequent deterioration in comfort. For example, in the case of a lower limb amputee, this can lead to the patient's inability to walk with a lower limb prosthesis.

Our convalescent ward accepted many amputees for prosthetic rehabilitation. Here, we present two cases of bone shortening of the stump that we have experienced.

## Case presentation

Case 1

**Figure 1:**
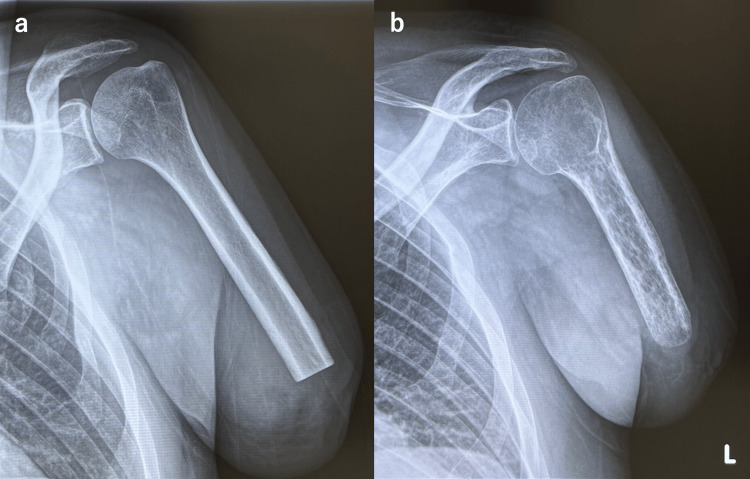
Plain radiographs of the stump in Case 1. (a) One month after surgery. The humeral length is 18.8 cm. (b) Nine months after surgery. The humeral length is 15.3 cm. The humeral length was measured along a straight line from the greater tubercle to the distal end of the humerus.

Case 1 involved a 57-year-old male. He was working in a metalworking factory when his left upper extremity was caught in a conveyor belt. Immediately after the injury, he was transported to an emergency medical center, where he underwent transhumeral amputation because it was difficult to salvage the limb. His only pre-existing medical history was hypertension. Approximately one month after surgery, he visited our outpatient clinic for upper limb prostheses. He underwent body-powered upper limb prosthetic rehabilitation while attending an outpatient clinic, and the prosthetist in charge fabricated a body-powered upper limb prosthesis. Nine months postoperatively, plain radiography was performed to assess the condition of the stump, which revealed bone shortening of the humerus at the stump. The humeral length had shortened by 3.5 cm compared with a plain radiograph taken one month postoperatively (Figure 1). The patient's stump remained free from bacterial infections throughout this period.

Case 2

**Figure 2:**
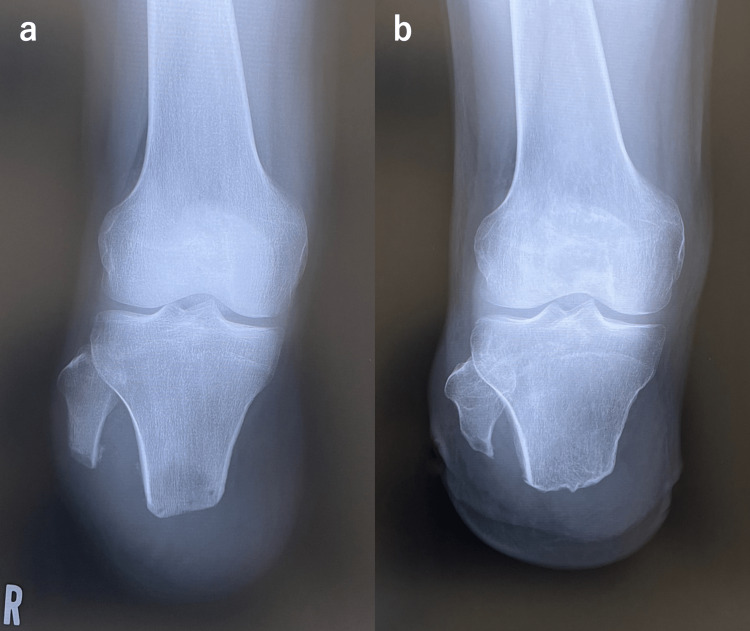
Plain radiographs of the stump in Case 2. (a) One month and a half after surgery. The tibial length is 8.9 cm. (b) Seventy-four months after surgery. The tibial length is 7.5 cm. The tibial length was measured straight from the medial tibial plateau to the distal end.

Case 2 involved a 27-year-old male. After a train accident, he underwent right transtibial amputation in an emergency medical center. He also experienced a traumatic subarachnoid hemorrhage at the same time but recovered without sequelae. He had no pre-existing medical history. He was admitted to our convalescent rehabilitation ward 1.5 months after surgery and underwent prosthetic rehabilitation. The patient was discharged home four months postoperatively after being able to walk with his lower limb prosthesis. Plain radiographs of the stump were taken at six years and two months postoperatively during outpatient visits. The tibial length had shortened by 1.4 cm compared with a plain radiograph taken 1.5 months postoperatively (Figure 2). The patient's stump has remained free from bacterial infections throughout these six years.

## Discussion

We report two cases of shortening of the residual bone after amputation. The two patients were of different ages and amputation sites, but the cause of amputation was the same: trauma. The degree of residual bone shortening varied from 1.4 to 3.5 cm (Table 1). We cannot determine whether 1.4 cm is long or short because no previous reports exist. This degree of shortening of the stump bone has also not been reported in the past; we report it here because 1.4 cm is a significant change to fit a prosthesis.

**Table 1:** Patient characteristics and the length of bone shortening at the stump.

Case	Age	Sex	Amputation level	Cause for amputation	Elapsed time since the initial X-ray (months)	Length of bone shortening (cm)
1	57	Male	Transhumeral	Trauma	8	3.5
2	27	Male	Transtibial	Trauma	72	1.4

A previous study reported shortening of the residual bone at the stump [4]. This case report describes a 39-year-old female with juvenile idiopathic arthritis who underwent transfemoral amputation due to infection after total knee arthroplasty and showed postoperative bone shortening of the stump. The patient had also undergone a previous total hip arthroplasty, and cement remained on the bony edge of the stump. Bone shortening of 2 cm around the cement was observed one year postoperatively. No postoperative infection occurred in the stump. The authors are unsure of the exact cause but hypothesize that the patient's inflammatory disease and inadequate mechanical loading of the amputation edge may have had something to do with it.

However, we do not necessarily agree with the theory that bone shortening after amputation is related to the patient's history of the inflammatory disease since both patients we presented here had amputation caused by trauma and had no underlying disease other than hypertension. The two cases we present here suggest that the bone at the stump may be idiopathically shortened without any particular cause.

Prosthetic users often place more weight on the non-amputated side than on the amputated side [5,6]. Consequently, the knee and hip joints on the non-amputated side are more prone to osteoarthritis, and the bone mineral density of the femoral neck on the amputated side tends to be lower [5-7]. Plain radiographs of the transhumeral amputee in Case 1 showed marked bone atrophy, possibly due to insufficient loading on the amputation. Similar to the reduction in bone density, we hypothesize that shortening of the stump end could be due to decreased vertical loading on the stump bone. The two cases we studied involved using upper and lower limb prostheses. Prosthetists generally fabricate them to minimize vertical pressure on the stump bone. Thus, this fabrication method reinforces the theory that the lack of vertical loading on the bone ends of the stump led to significant bone resorption and shortening. However, this hypothesis does not account for why we did not observe similar changes in bone length in other amputees.

This case report suggests that shortening of the residual bone at the stump can occur but may go unnoticed. However, the results of this case report do not provide clear evidence of the causes, trends, or mechanisms of bone shortening. Therefore, further data collection and basic medical research are needed to determine the cause of postamputation bone shortening.

## Conclusions

We reported two cases of bone shortening at the stumps after amputation. The causes of amputations in these patients were trauma. It is possible that bone shortening at the stumps has gone unnoticed until now, even though it has occurred. However, the underlying mechanism remains unclear, and further studies are required.
